# Multi-cancer early detection (MCED) tests: prioritizing equity from bench to bedside

**DOI:** 10.1093/haschl/qxae039

**Published:** 2024-05-23

**Authors:** Sarah J Miller, Jamilia R Sly, Christian Rolfo, Philip Mack, Augusto Villanueva, Melissa Mazor, Ellerie Weber, Jenny J Lin, Cardinale B Smith, Emanuela Taioli

**Affiliations:** Department of Population Health Science and Policy, Icahn School of Medicine at Mount Sinai, New York, NY 10029, United States; Department of Family Medicine and Community Health, Icahn School of Medicine at Mount Sinai, New York, NY 10029, United States; Tisch Cancer Institute, Icahn School of Medicine at Mount Sinai, New York, NY 10029, United States; Department of Population Health Science and Policy, Icahn School of Medicine at Mount Sinai, New York, NY 10029, United States; Department of Family Medicine and Community Health, Icahn School of Medicine at Mount Sinai, New York, NY 10029, United States; Tisch Cancer Institute, Icahn School of Medicine at Mount Sinai, New York, NY 10029, United States; Tisch Cancer Institute, Icahn School of Medicine at Mount Sinai, New York, NY 10029, United States; Center for Thoracic Oncology, Icahn School of Medicine at Mount Sinai, New York, NY 10029, United States; Tisch Cancer Institute, Icahn School of Medicine at Mount Sinai, New York, NY 10029, United States; Center for Thoracic Oncology, Icahn School of Medicine at Mount Sinai, New York, NY 10029, United States; Tisch Cancer Institute, Icahn School of Medicine at Mount Sinai, New York, NY 10029, United States; Mount Sinai Liver Cancer Program, Division of Liver Diseases, Icahn School of Medicine at Mount Sinai, New York, NY 10029, United States; Tisch Cancer Institute, Icahn School of Medicine at Mount Sinai, New York, NY 10029, United States; Division of General Internal Medicine, Icahn School of Medicine at Mount Sinai, New York, NY 10029, United States; Department of Population Health Science and Policy, Icahn School of Medicine at Mount Sinai, New York, NY 10029, United States; Tisch Cancer Institute, Icahn School of Medicine at Mount Sinai, New York, NY 10029, United States; Division of General Internal Medicine, Icahn School of Medicine at Mount Sinai, New York, NY 10029, United States; Tisch Cancer Institute, Icahn School of Medicine at Mount Sinai, New York, NY 10029, United States; Brookdale Department of Geriatrics and Palliative Medicine, Icahn School of Medicine at Mount Sinai, New York, NY 10029, United States; Department of Population Health Science and Policy, Icahn School of Medicine at Mount Sinai, New York, NY 10029, United States; Tisch Cancer Institute, Icahn School of Medicine at Mount Sinai, New York, NY 10029, United States; Department of Thoracic Surgery, Icahn School of Medicine at Mount Sinai, New York, NY 10029, United States; Institute for Translational Epidemiology, Icahn School of Medicine at Mount Sinai, New York, NY 10029, United States

**Keywords:** health equity, MCED, cancer screening, liquid biopsy

## Abstract

Multi-cancer early detection (MCED) tests are blood-based tests designed to screen for signals of multiple cancers. There is growing interest and investment in examining the potential benefits and applications of MCED tests. If MCED tests are shown to have clinical utility, it is important to ensure that all people—regardless of their demographic or socioeconomic background—equitably benefit from these tests. Unfortunately, with health care innovation, such considerations are often ignored until after inequities emerge. We urge for-profit companies, scientists, clinicians, payers, and government agencies to prioritize equity now—when MCEDs are still being developed and researched. In an effort to avoid creating and exacerbating cancer inequities, we propose 9 equity considerations for MCEDs.

## Cancer screening

Cancer is the second leading cause of death in the United States and more than 600 000 people die each year from the disease.^1^ Cancer screening tests can aid in the early detection and prevention of cancer. The US Preventive Services Task Force (USPSTF) outlines Grade A and B recommendations for breast, cervical, colorectal, and lung cancer screening.^2^ Unfortunately, more than half of cancer-related deaths are from cancers that do not have a recommended screening test.^1^

In 2022, President Biden reignited the Cancer Moonshot initiative and set an ambitious and bold goal to reduce the cancer death rate by half within the next 25 years.^3^ Consequently, Drs. Knudsen and Febbo^4^ maintained that, “Cutting cancer deaths in half will require bucking the status quo and developing more effective, accurate, and comprehensive screening approaches on an accelerated timeline. There is an urgent need to develop effective strategies for the currently ‘non-screenable’ cancers….” There is growing interest in examining the potential use of other screening methods, including liquid biopsy, to aid in the early detection of cancer.

## Multi-cancer early detection tests

Liquid biopsy^5^ holds promise to revolutionize and enhance our approach to cancer screening. The term “liquid biopsy” can refer to cancer detection using any biological fluid (including saliva, urine, cerebrospinal fluid, and many others), but is most typically carried out in blood draws. Liquid biopsy entails the analysis of tumor components released into these body fluids, including circulating tumor cells, proteins, or fragments of tumoral DNA or RNA. These tumor-specific components may be freely circulating or packaged in tiny extracellular vesicles that can be extracted from blood. The liquid biopsy market size is expected to grow almost 5-fold in the next 10 years (from $4 billion in 2022 to a projected $18 billion in 2032).^6^

Research is exploring using liquid biopsy to screen for multiple cancers in parallel, as it is relatively routine and minimally invasive to draw blood. These multi-cancer early detection (MCED) tests are designed to detect signals for cancer in asymptomatic individuals who have not already been diagnosed with cancer. Multi-cancer early detection tests can serve as a complement to existing single-cancer screening tests or potentially as a screening option for cancers that do not have USPSTF-recommended screening approaches.^7,8^ In either context, these MCED tests will need to be extraordinarily sensitive (able to identify exquisitely minute quantities of the tumor-specific target), while maintaining a very high specificity (reducing false-positive errors). One highly promising application is to investigate changes in DNA methylation that are caused by the tumor process. These changes can be identified in tiny fragments of tumor DNA that are shed into the bloodstream.

Emerging research has begun to evaluate these MCED tests. For example, preliminary results of the PanSeer study^9^ demonstrated the potential of the DNA methylation approach to screening using blood samples. The investigators drew blood samples from a large number of healthy individuals and then focused on patients who were later diagnosed with cancer. They detected evidence of cancer in over 90% of these cases, in blood samples acquired before diagnosis. While more evidence regarding the clinical utility of the test is required before this test can be used routinely, proof-of-principle was established.

In addition, in the DETECT-A (Detecting cancers Earlier Through Elective mutation-based blood Collection and Testing) study, researchers followed approximately 10, 000 women with no prior history of cancer, with a particular focus on feasibility and safety of a blood-based screening approach. The investigators reported that, of 134 participants who maintained a positive signal on a second blood draw, 127 had subsequent imaging. Of these, 64 patients (50%) had results suggestive of cancer, with 26 patients having a proven cancer. An additional 24 patients from the original cohort were also determined to have or develop cancer, but with no blood finding.^10^ A follow-up study^11^ of 98 patients with an initial false-positive blood finding found that almost all (96) remained free of cancer, serving as both a cautionary note on assay performance and a positive note on the efficacy of follow-up imaging.

Furthermore, the results obtained from the PATHFINDER study (NCT04241796) have been recently released. The prospective cohort enrolled 6662 adults without clinical suspicion of cancer. Among the sample, a cancer signal was detected in 92 (1.4%), of the patients, of whom 35 (38%) were later diagnosed with cancer and 57 (62%) were not diagnosed with cancer. The median time to diagnostic resolution was 79 days (median of 67 days in true positives and 162 days in false positives).^12^

Although the initial research surrounding MCED tests is promising, there are still several unanswered questions about the efficacy, effectiveness, clinical utility, and potential clinical application of MCED tests.^13^ Research is still needed to elucidate whether MCED tests reduce cancer mortality and to identify potential downstream drawbacks associated with the tests (eg, over-screening, overdiagnosis, false positives, false negatives). Such studies can inform guidelines about the appropriate clinical use of these tests (eg, which patients should be screened, frequency of screening, recommended follow-up testing). Several ongoing and future studies plan to rigorously answer these questions.^14^

## MCED tests and equity

Although cancer can affect everyone, some groups shoulder a greater burden of the disease, including minoritized populations (ie, individuals who have been actively minoritized due to social factors including racism and structural exclusion^15^). According to the American Association for Cancer Research (AACR) Cancer Disparities Progress Report, minoritized people and historically underserved groups (ie, people with low socioeconomic status, rural communities, and gender and sexual minorities) are disproportionately impacted by cancer and its sequalae.^16^

Far too often, individuals with financial resources and structural privilege are the first to access and benefit from medical innovations. Historically underserved and minoritized groups, on the other hand, are often systemically excluded from accessing cutting-edge medical care. The Inverse Care Law, a concept that was introduced in 1971,^17^ describes the medical injustice that occurs when good medical care is inversely related to need for that care in a population. It is critically important to keep an eye on equity and to ensure that all people—regardless of their financial and social capital—are able to benefit from all emerging medical innovations. With regard to MCED tests, it is crucial to identify whether and how these tests could impact equity, in order to prevent creating and/or exacerbating disparities in cancer.

Unfortunately, equity considerations are often addressed too late, after inequities have already emerged. We urge for-profit companies, scientists, clinicians, payers, and government agencies to prioritize equity now—as MCED tests are still being developed and researched. These considerations are essential across all stages of research and clinical practice—from initial clinical trials to future dissemination. We posit that equity should be at the forefront of decision making, while research is ongoing. The purpose of this article is not to comment on the benefits or drawbacks of MCED but rather to highlight important equity considerations that should be central to ongoing research and dissemination efforts.

## Equity considerations with MCEDs

### Representation and diversity in clinical trials

It is important that MCED clinical trials enroll diverse, heterogeneous samples. It is particularly pressing to include participants who have higher cancer incidence, morbidity, and mortality rates. Diverse samples can allow for subgroup analyses to determine whether the efficacy of the MCED test differs based on individuals’ demographic background (eg, ancestry, sex assigned at birth), genetic makeup, and environmental exposures. Such information can guide decision making regarding the best clinical application of the MCED test for higher-risk populations. Furthermore, heterogeneous enrollment in clinical trials maximizes the generalizability and potential impact of the results.

In fact, in the PATHFINDER study,^12^ the sample was predominantly non-Hispanic White (91.7%). Similarly, in the DETECT-A study,^10^ the majority (∼95%) of the sample was non-Hispanic White. Unfortunately, this problem is not unique to MCED-related trials.^18^ Clinical trials with homogenous, predominantly White participants will likely decrease patient trust and acceptance of the novel technologies being tested, particularly if patients’ demographic backgrounds are not represented in the trials. In fact, leading governmental and funding agencies deem diversification of clinical trial representation a high priority to achieve care equity.^19^

#### Recommendation

Researchers and funders should prioritize enrollment of diverse heterogeneous cohorts in MCED trials. Published guidelines and recommendations for improving diversity in clinical trials should be followed (eg, reference 20-22). For example, the MCED Consortium is a US/UK consortium that aims to evaluate MCED tests and offer guidance on potential future implementation. As part of the consortium, an MCED equity working group offered recommendations for ways to increase diversity in MCED clinical trials.^23^ Multilevel recommendations to increase diversity were outlined and include the following: investigator-level measures (eg, mitigating provider bias), participant-level measures (eg, understanding motivators of patient participation in clinical trials), underrepresented participant measures (eg, partnering with communities of color including advocacy groups, community-based organizations), and study measures (eg, setting broad enrollment areas).

With regard to MCED trials specifically, it is also important to ensure that participants do not incur costs associated with study participation (eg, transportation, time off work) and from any resultant follow-up testing and treatment.

### Equitable insurance coverage and access to MCED tests

As evidence continues to emerge about the potential benefits of MCED tests, and we possibly move toward clinical implementation, it is important to ensure that all patients—regardless of their insurance coverage—have access to these potentially lifesaving tests, if they are shown to have clinical utility. Currently, MCED tests are not covered by insurance and thus may be limited to those who can pay for the out-of-pocket expenses for these tests (eg, the Galleri test is $949 for self-pay patients).^24^ If ongoing trials prove the clinical utility of MCED tests and these tests receive Food and Drug Administration (FDA) approval, insurance coverage for them will likely expand. In anticipation of this, federal legislators have introduced the Nancy Gardner Sewell Medicare Multi-Cancer Early Detection Screening Coverage Act/Medicare Multi-Cancer Early Detection Screening Coverage Act, which would authorize the Centers for Medicare and Medicaid Services to evaluate and cover MCED tests if FDA approval is achieved.^25^ Such legislation would also ensure that the many socioeconomically disadvantaged patients receiving Medicare have access to these tests. However, at the time of this writing, the legislation has not been approved by Congress.

#### Recommendation

Policymakers should advocate for inclusive coverage of these tests in order to avoid possible delays and gaps in coverage if MCED tests receive FDA approval. In addition, policymakers should advocate for both national- and state-level programs to expand screening access to include MCED tests for those who are uninsured and underinsured, as has been done with other cancer screenings (eg, NY State Cancer Services Program^26^).

### Equitable coverage for necessary diagnostic follow-up testing

Equitable access to cancer care does not end with access to cancer screenings. It is important that payers expand coverage for not only cancer screening but also for any diagnostic follow-up testing required after abnormal results from an MCED test. This is an especially vital consideration in the context of research demonstrating that, even when cancer screening tests are free, the diagnostic follow-up testing can be costly.^27-29^ Research has shown that financial toxicity resulting from copayments, co-insurance, and deductibles can lead patients to not take their medications as directed (in order to save money on copayments), have a lower quality of life, and face debt/bankruptcy.^30^ Cancer screenings are only effective when necessary follow-up diagnostic testing and treatment are completed. Of great concern, the out-of-pocket costs associated with these follow-up tests could be financially devastating, particularly for low-income or low-resourced patients.

#### Recommendation

If research proves the clinical utility of MCED tests and these tests receive FDA approval, payers and policymakers should also expand coverage of necessary follow-up testing. Of relevance, with stool-based colorectal cancer screenings, payers are mandated to cover necessary follow-up testing to complete the screening (ie, colonoscopy following an abnormal stool-based test).^31^ Policymakers and payers could consider a similar model for MCEDs.

### Equitable and meaningful access to MCEDs

Access to MCEDs is not limited to insurance coverage. Addressing financial barriers is necessary, yet insufficient to ensure equitable access to MCED tests. Many people in the United States, particularly minoritized and historically underserved patients, do not have access to consistent health care. It is estimated that over 100 million individuals living in the United Sates are “medically disenfranchised,” meaning they do not have regular access to primary care. More than half of the people who are deemed medically disenfranchised are at or below 200% of the federal poverty level.^32^ Therefore, equitable access to MCED tests must expand beyond the clinic doors.

#### Recommendation

If MCED tests receive FDA approval and their clinical utility is established, companies selling MCED tests should partner with minority-serving health care systems, including federally qualified health centers and safety-net hospitals, to make the tests available to all patients. Furthermore, it is important that access is not limited to traditional health care settings. Mobile phlebotomy units, for example, can be leveraged to offer these potentially lifesaving tests to patients in their communities. Community-based outreach and access are critically important, particularly for the more than 100 million Americans who are medically disenfranchised.

### Comprehensive navigation services

It is important to provide patients with support services to help navigate the health care system, particularly when diagnostic testing and cancer treatment are necessary following MCED tests. Decades of research have found that patient navigation can help patients who are adversely affected by health inequities complete important cancer screenings.^33^ Navigators can help individuals overcome system-level barriers to care (eg, transportation, childcare, appointment scheduling) as well as individual barriers (eg, low health literacy, ambivalence/uncertainty about getting screened) and improve access to care and quality of life.^34^

#### Recommendation

Researchers should conduct rigorous mixed-methods studies to identify the patient- and system-level barriers to completing MCED testing as well as barriers to completing any necessary following up diagnostic testing. Drawing from the broader patient navigation literatures, patient navigation programs could be developed to help patients overcome these identified barriers. Health care systems, payers, and for-profit MCED companies should allocate funds to pay for these navigation programs.

### Accessible education and outreach

If MCED tests’ clinical utility is established and they become a part of standard clinical care, it will be necessary to educate the public about the potential benefits, risks, and appropriate uses of these tests. However, education efforts should not be limited to marketing efforts about the availability of the test, but rather should offer a comprehensive explanation of the test and how it may impact lives, particularly for people with fewer financial and social resources. For example, individuals should understand the full financial obligations that may be associated with the MCED tests and, if necessary, follow-up testing. From a psychological standpoint, it is also important that people understand the implications of having an abnormal result on an MCED test, including a nuanced understanding of the potential for false-positive results or false-negative results. Although the data are mixed, there is evidence that false positives on cancer screenings can negatively impact patients’ mental health and quality of life.^35^

#### Recommendation

Researchers, clinicians, and for-profit companies should collaborate with community groups to develop and test robust education materials to educate the public about MCED tests. Given the complexity surrounding the potential costs/benefits of the tests, a shared decision-making approach can help patients develop a nuanced understanding of these tests and make an informed decision about whether or not to have the test, as well as accurate interpretation of the results. All shared decision-making and educational efforts must be accessible and understandable for individuals with varying levels of health literacy and general literacy. In addition, it is important that information about these innovative tests is delivered in multiple languages so that non–English-speaking patients have access to the information. The Agency for Healthcare Research and Quality's SHARE Approach Curriculum Tool^36^ offers guidance and recommendations for implementing shared decision making in a variety of setting and contexts (eg, working with patients with varying levels of health literacy). If the clinical utility of MCED tests is established and agencies (eg, American Cancer Society, USPSTF) ultimately recommend MCED tests for clinical use, it may be beneficial to consider recommendations for shared decision making before completing an MCED test (similar to prostate cancer screening recommendations).^37^ Recent evidence^38,39^ shows that most patients and providers are receptive to MCED tests and clinical trial participation however, barriers may exist (eg, worry about costs, concerns about test accuracy).

### Community trust

Medical mistrust is a pervasive and growing problem in the US health care system. Several multilevel factors (individual, institutional, structural), including racism, discrimination, bias, medical exclusion, and proliferation of misinformation, have fueled mistrust in medicine, research, and health care. Such mistrust has been exacerbated in recent years, particularly in the context of COVID-19 misinformation and the relatively recent spotlight that has been cast on health inequities and medical racism.^40-42^

MCED tests may face unique challenges with medical mistrust, in the context of the widely publicized Theranos medical fraud case.^43,44^ Theranos claimed to be able to run multiple tests from a single drop of blood. The potential impact of the Theranos case on MCED acceptance has not been studied and the negative consequences on patient perceptions of MCED tests are unknown. Future research is needed to understand the factors that may influence perceptions of MCED tests.

#### Recommendation

Health care systems, including academic researchers, must work to earn the public's trust, particularly among minoritized and historically underserved patient populations. We maintain that it is the health care system's responsibility, not the patients', to repair previous harm and earn community trust. We posit that the key to building and sustaining community trust lies in fostering meaningful and mutually beneficial community partnerships. In their paper on building community trust, Christopher et al^45^ outline key aspects of building trust, including the following: “(1) acknowledge personal and institutional histories, (2) understand the historical context of the research, (3) be present in the community and listen to community members, (4) acknowledge the expertise of all partners, and (5) be upfront about expectations and intentions.” In our experience, community partnerships can take years to develop and sustain. As such, it is important to start fostering these relationships now, in anticipation of potential future MCED dissemination.

### Diverse and inclusive workforce

A diverse workforce can foster improved science, innovation, and health outcomes. With regard to the development, testing, and possible future dissemination of MCED tests, it is critical that a diverse workforce is leading the charge. Diverse and inclusive teams—including policymakers, researchers, for-profit companies, and clinicians—can help ensure that new technologies are developed and disseminated in an equitable manner and can help avoid unintentionally creating or exacerbating health disparities.

#### Recommendation

Policymakers, researchers, for-profit companies, funding agencies, and health care teams should strive to have diverse teams and heterogeneous perspectives leading decision making about developing, testing, and possibly implementing MCED tests.

### Avoidance of downstream system burden

As discussed above, if the clinical utility of MCED tests is established and these tests are disseminated widely into standard clinical care, it is important to consider potential unintended consequences. Of particular concern, as more patients are screened with MCED tests, more patients will require follow-up diagnostic testing. We must ensure that the health care system is prepared to handle the potential increase in demand for testing. An increased demand may lead to delays in cancer diagnoses for all patients. It is important that the health care systems, particularly underresourced clinics and hospitals, are adequately prepared to handle the increase in demand. Of great concern, some evidence has shown that patients of color already experience longer wait times with regard to cancer-related care.^46^ Therefore, it is critical to avoid perpetuating and perhaps exacerbating these disparities.

#### Recommendation

It is premature to make decisions regarding the clinical application of MCED tests (eg, test/time intervals, targeted age groups) before the clinical utility has been established. However, without this information, the potential impact of the MCED tests on downstream system-level burden is unknown. Therefore, it is important that prospective research studies examine the impact of MCED tests on health care systems as a key outcome. Additionally, modeling studies can predict potential downstream impacts on the health care systems. These data will be essential to help health care systems actively prepare for potential changes in the demand.

## Conclusion

If the evidence supports the clinical utility of MCEDs, we may see a dramatic shift in cancer-screening practices in the near future. These tests have the potential to vastly expand our ability to detect cancer, particularly among currently “unscreenable” cancers. Despite the promise of these tests, enthusiasm is tempered because there are still many unanswered questions about their clinical utility. Far too often, equity is an afterthought. Therefore, as with all emerging innovations, it is critical to prioritize equity early on. We urge policymakers, payers, health care providers, funding agencies, and for-profit companies to use our recommendations to prioritize equity in the testing and potential rollout of MCED tests in a comprehensive effort to provide the highest quality care to all patients. We posit that MCED stakeholders need to work collaboratively to avoid a potential diffusion of responsibility. Without a collaborative and comprehensive plan to address inequities, we may witness the creation and widening of inequities in cancer outcomes.

## Supplementary Material

qxae039_Supplementary_Data
